# A Novel RNF139 Mutation in Hemangioblastomas: Case Report

**DOI:** 10.3389/fneur.2019.00359

**Published:** 2019-04-12

**Authors:** Ping Yang, Liang Li, Wei Zhang, Bo Liu, Ling Li, Hongxing Huang, Kun Liu, Hua Liu, Huiyong Huang, Feng Li, Shucheng Zou

**Affiliations:** ^1^Department of Neurosurgery, Hunan Brain Hospital, Clinical Medical School, Hunan University of Chinese Medicine, Changsha, China; ^2^Provincial Key Laboratory of TCM Diagnostics, Hunan University of Chinese Medicine, Changsha, China; ^3^The Discipline of Chinese and Western Integrative Medicine, Hunan University of Chinese Medicine, Changsha, China; ^4^School of Dentistry, University of California, Los Angeles, Los Angeles, CA, United States

**Keywords:** hemangioblastomas, RNF139, mutation, next-generation sequencing, sanger sequencing

## Abstract

Hemangioblastomas (HBs) are classified as grade I tumors with uncertain origin according to the World Health Organization's classification system. HBs are characterized by rich mesenchymal cells and abundant capillaries. It has been shown that tumorigenesis of HBs depends on mutational inactivation of Von Hippel-Lindau (VHL) tumor suppressor gene. Therefore, the majority of patients will undergo VHL single gene test, and sequencing scheme is rarely used in clinic. In this study, we described a girl and her father successively found to have HBs within half a year. The results of next-generation sequencing (NGS) and Sanger sequencing analysis showed that both of them carried heterozygous mutation of RNF139 p.Q650R. This mutation was interpreted as Pathogenic variation based on the American College of Medical Genetics and Genomics (ACMG) guideline. Sanger sequencing was performed with other family members. No mutation on rs118184842 locus of RNF139 gene was found in the samples from the girl's mother, uncle and aunt. This report supports that the novel mutation of RNF139 p.Q650R probably serve as a key role in HBs progression.

## Introduction

Central nervous system hemangioblastomas (HBs) are benign tumors which usually occur in cerebellum (1), and the pathogenesis of HBs is still unknown. According to the revised World Health Organization (WHO) classification of tumors of the nervous system published in 2000, HBs were classified to the tumors of uncertain histogenesis. The morphology code of the International Classification of Diseases for Oncology (ICD-O)is 9161/1, grade I. HBs are composed of mesenchymal cells and abundant capillaries, so it can also be called as capillary HBs. Some patients are accompanied with Von Hippel-Lindau (VHL) disease (2). VHL, an autosomal dominant hereditary disease, is one of the familial neoplastic syndromes affecting the nervous system (3). It has a broad spectrum of clinical manifestation, in which about 40 different lesions in 14 organs have been described. It is characterized by the occurrence of retinal or central nervous system HBs, renal clear-cell carcinoma, pheochromocytoma, pancreatic, and internal auditorytumors (4, 5), resulting from the germline mutation of the VHL tumor suppressor gene located on chromosome 3P25-26. The prevalence of VHL ranges from 1: 36,000 to 1: 45,500 (6). Males and females are equally affected.

In clinic, HBs patients often show progressively increasing intracranial pressure with unilateral cerebellar dysfunction, such as headache, ataxia, nausea, vomiting, dizziness, nystagmus, etc. A small proportion of cases may suffer erythrocythemia. These disorders can be cured by surgical removal. Approximately 25% HBs relapse after surgery and most of them are patients with VHL disease. The RNF139 gene has not previously been reported to be associated with HBs. In the past, this gene was found to be related to renal and tongue cancer. Although some researchers declared recently that abnormal expression of RNF139 protein was found in neurogliocytoma, no studies have been conducted using next-generation sequencing (NGS).

In this study, we described a 19-year-old girl with the history of headache, nausea and vomiting. A novel mutation in rs118184842locus of the RNF139 gene was found using NGS (Illumina, San Diego, California, USA), while there is not any mutation sequenced in VHL gene. The mutation was inherited from her father who had the same symptoms half a year later. We performed Sanger sequencing (Thermo Fisher Scientific, Waltham, Massachusetts, USA) on their families. The results showed that the girl's mother, uncle, and aunt had no mutation in this locus which was all in the wild type. It suggested that there was a correlation between RNF139 gene and the incidence of HBs.

## Case Report

### Case 1

**Figure 1:**
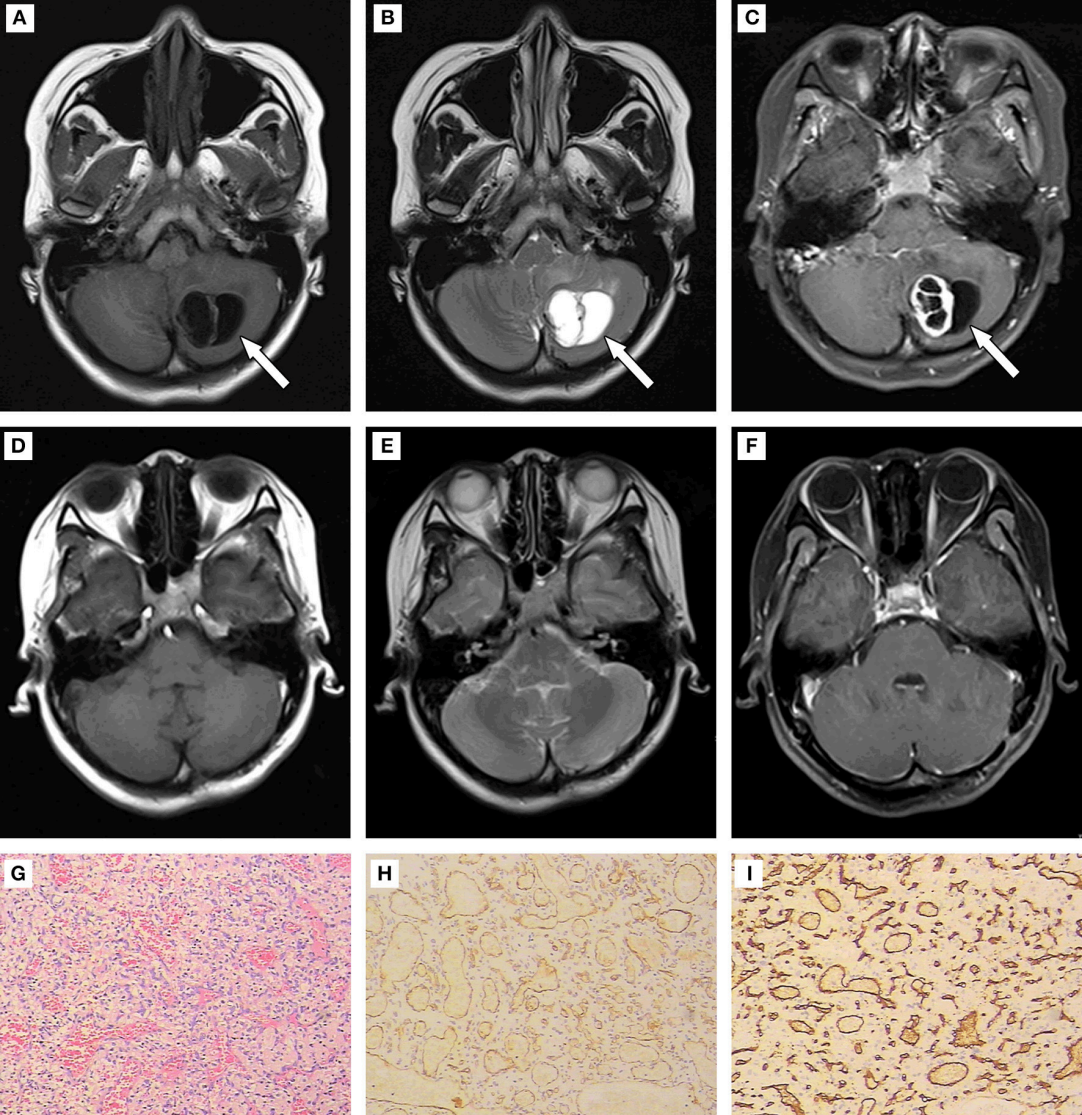
**(A–C)** Results of preoperative MRI. Round long T1 and long T2 signal intensities existed in the left cerebellar hemisphere. Iso-T1 and T2 signal intensities of septa were found inside. Enhanced scanning showed annular enhancement in solid portion and no enhancement in cystic portion of the mass. The fourth ventricle was compressed rightward, and became narrow. **(D–F)** Results of postoperative MRI. The patchy slight enhancement of the left cerebellar hemisphere was in line with the postoperative changes in tumor resection. **(G)** (200×): HE staining to tumor showed disorderly arrangement of cells. **(H,I)** (400×): Positive expression of CD 31 and CD 34 detected using immunohistochemistry.

A 19-year-old female kindergarten teacher presented with a 2-month history of repeated occipital headache which aggravates on activities, nausea and vomiting. When she was admitted to our hospital, no obvious neurological abnormalities were found via physical examination. The head magnetic resonance imaging (MRI, Siemens, Munich, Germany) scan showed a mass in the left cerebellar hemisphere (Figures 1A–F). The patient underwent the left cerebellar hemisphere tumor resection, dural repair, and cranioplasty 1 week later. Hematoxylin and Eosin (H&E) staining was performed (Figure 1G), and positive expression of CD31 and CD34 (Aotang Medical Technology Co., Ltd., Zhongshan, China) were detected using immunohistochemistry (Figures 1H,I). The postoperative pathological analysis and diagnosis confirmed the diagnosis of HBs.

### Case 2

**Figure 2:**
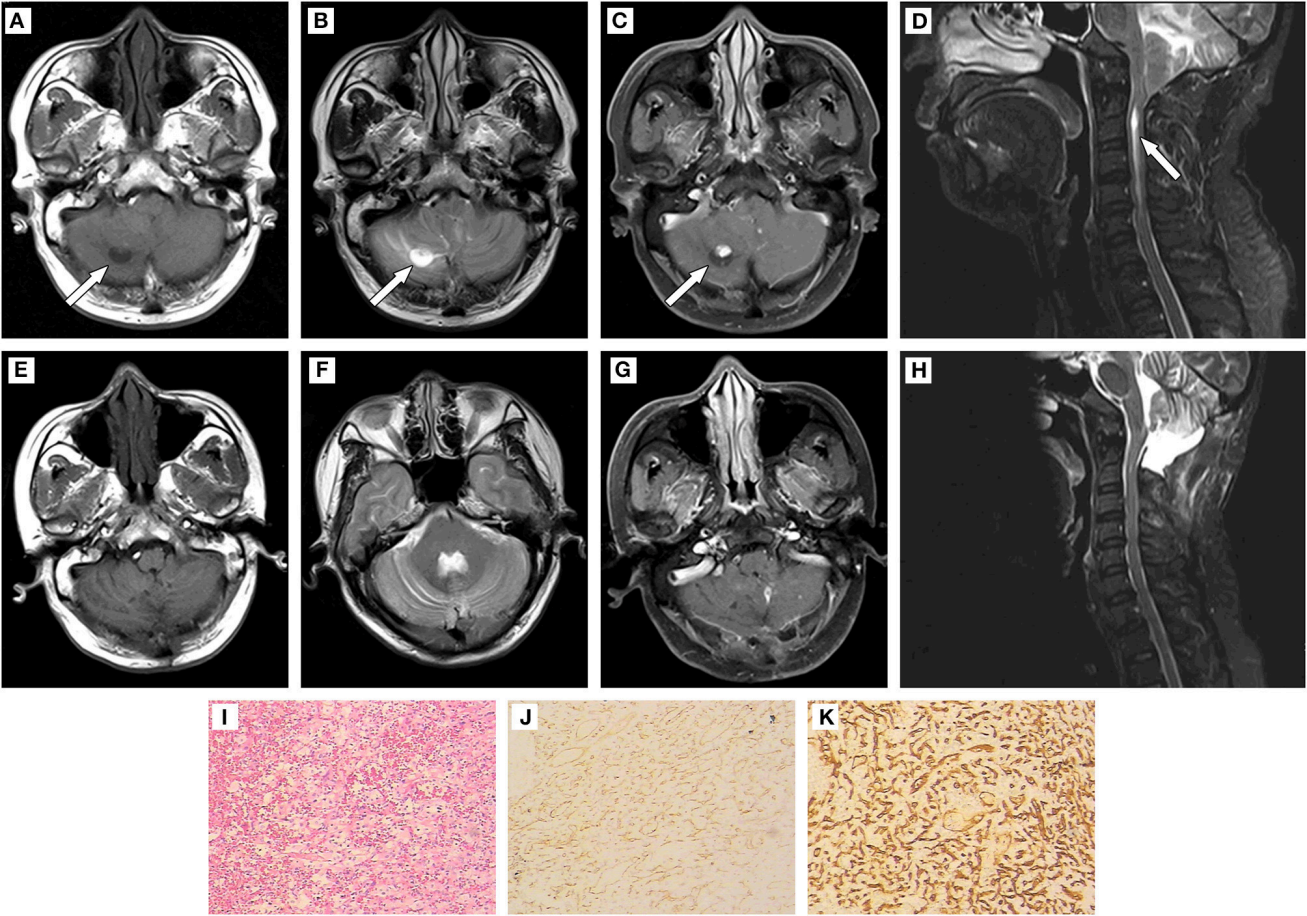
**(A**–**D)** Results of preoperative MRI. Patchy long T1 and long T2 signal intensities existed in the bilateral cerebellar hemispheres and vermis. Solid/cystic confounding signal intensities were observed in the enhanced lesion of right cerebellar hemisphere. The solid portion showed iso-T1 and T2 signal intensities, while cystic portion was long T1 and long T2 signal intensities. Enhanced scanning showed remarkable enhancement in solid portion and no enhancement in cystic portion of the mass. The brain stem was compressed, and the fourth ventricle became narrow. The supratentorial ventricle system expanded, with the tonsillar hernia intruded into the spinal canal. The central canal of the cervical cord expanded and strip-shaped long T1 and long T2 signal intensities was found at the level of C2-C6. **(E–H):** Results of postoperative MRI. The initial lesion signals reduced. The solid/cystic lesions in the right cerebellar hemisphere and punctate enhancement lesions in the left cerebellar hemisphere were not shown clearly. The fourth ventricle enlarged. The cerebellar tonsillar did not develop into the spinal canal. A strip-shaped long T1 signal intensity still existed in the cervical cord. **(I)** (200×):HE staining to tumor showed disorderly arrangement of cells. **(J,K)** (400×): Positive expression of CD 31 and CD 34 detected using immunohistochemistry.

The girl's father, a 44-year-old peasant, suffered from a headache 6 months later, and the right occipital swelling pain was the main symptom without nausea and vomiting. Physical examination showed no abnormal neurological symptoms. Results of MRI indicated multiple masses in the cerebellum and spinal cord (Figures 2A–H). Multiple cysts were found on the right kidney with B-mode ultrasound. The patient underwent the multiple cerebellar tumor resections, posterior cranial fossa decompression, and dural expansion plasty 1 week after admission. The detection of H&E staining (Figure 2I) and immunohistochemistry (Figures 2J,K) was the same as Case 1. The postoperative pathological analysis and diagnosis confirmed the diagnosis of HBs.

### Genetic Analysis and Results

Totally 10 ml peripheral blood was collected from each patient and centrifuged to separate white blood cells. Genomic DNA was extracted and the standard library construction was performed followed by targeted sequencing. Probes were designed based on the Agilent platform (Agilent, Santa Clara, California, USA) and the entire exon regions of 140 brain disease-associated genes were extracted. The high-throughput sequencing was employed on the NGS platform with an average depth of more than 250×. Variant calling was completed using GATK 3.0 (7). All gene mutation results were visually measured and confirmed using the Integrative Genomics Viewer (8), and the pathogenicity classification was interpreted according to American College of Medical Genetics and Genomics (ACMG) guidelines (9). Pathogenic or Likely Pathogenic variants were further identified using Sanger sequencing for mutation determination and family validation.

**Figure 3:**
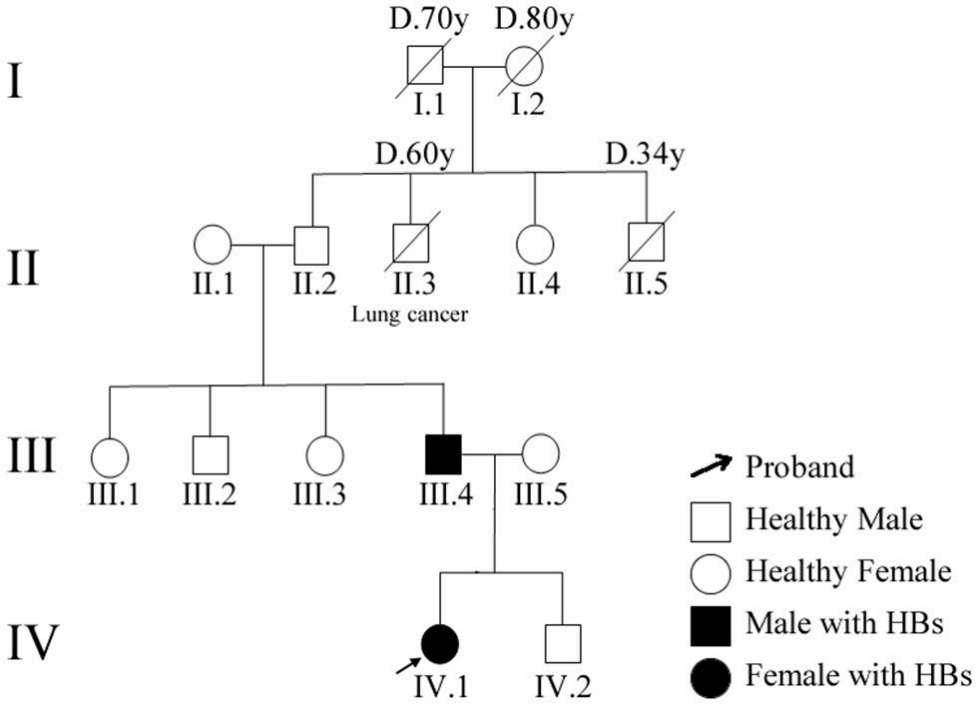
Patients' family pedigree for RNF139. Family numbers and disease-causing variant is noted above pedigree. Normal individuals are shown as clear circles (females) and squares (males). The patient above the arrow indicates the proband (IV.1).

**Figure 4:**
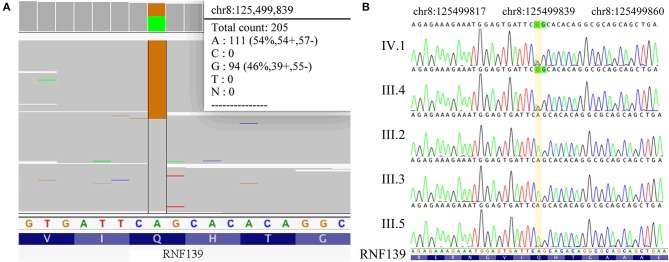
**(A)** Results of NGS (RNF139 p.Q650R). **(B)** Results of Sanger sequencing. The sequencing results in IV.1 and III.4 is RNF139 mutant homozygous type, while in III.2, III.3, and III.5 is wild type. The yellow region indicates the duplication at the nucleotide position for RNF139 gene NM_007218.

This family pedigree was shown in Figure 3. A total of 10 gene mutations with population frequency <0.5% were detected via targeted high-throughput sequencing. However, no VHL genes mutation was found in the sample, then we inferred that there might be some other important mutations, including heterozygous variants of A-TO-G located in rs118184842 locus of RNF139 gene. Due to such mutation, the Gln located on the 650 of exon 2 in RNF139 gene was replaced by Arg (the referred transcript number: NM_007218). Only this mutation was interpreted as pathogenic variation (PVS1, PS1) with ACMG guideline (Figure 4A). Family validation results using Sanger sequencing showed that only the girl and her father carried the mutation. Both of them suffered headache in the occipital area and the head MRI showed cerebellar space-occupying lesions. However, the father was found multiple lesions in the cerebellum and cervical cord as well as the right renal cyst. The locus of the girl's mother, uncle and aunt were all in the wild type (Figure 4B). They did not show any clinical symptoms and no abnormalities were found in head MRI.

## Discussion

Several investigations described the relationship of RNF139 gene with different cancers (10–12). A significant amplification of RNF139 was found in oral cancer (13), and RNF139 could regulate the progression of tongue cancer by adjusting AKT signal pathway. Chen et al. (14) identified RNF139 is one of the driver genes in melanoma, liver and breast cancers based on the computational analysis of large-scale cancer genomic datasets. These studies showed that RNF139 gene has a strong correlation with the occurrence and development of various cancers.

Gln650 in RNF139 is a highly conservative amino acid among species. The mutation of the Gln (uncharged polar amino acid) replaced by Arg (charged polar amino acid) could result in the polarity change in amino acid located on the 650 of exon 2 in RNF139 gene. Studies have reported that changes in amino acid polarity had serious effects on protein structure stability and function (15). According to the ACMG guideline, this mutation may affect the protein structure and function due to its pathogenic role.

Up to date, no clinical data has yet to confirm the correlation between the mutations of RNF139 and the occurrence of cancers. Although these two patients had the same mutation locus, there were more multiple lesions in case 2, especially in his cerebellum and cervicalcords, which brought more trouble to doctors in surgery. Both patients underwent lesions resection. The daughter recovered quickly and went to work 2 months later. The father still suffered from the spasmatic headache after surgery, and the symptom alleviated after 6 months. In spite of no relapse signs occurred during follow-up visits up to now, we still predict that the recurrence rate of the father may be higher than that of his daughter according to the father's imaging examination. These may indirectly support a pathogenic role of this novel mutation in cancer progression.

In this study, although we detected a total of 140 exon regions of brain disease-related genes using targeted sequencing, variations in introns and structural variations that were out of the extraction range can affect protein function. In addition, this result can't exclude the existence of important mutations in other undetected genes, which may affect the occurrence of the disease. The effect of RNF139 p.Q650R mutation on RNF139 function also remains to be determined in future studies. Nonetheless, this study is the first report about the HBs family. On the basis of the family validation and head MRI examinations on most members of this HBs family, we can conclude that RNF139 mutation is related to the pathogenesis of the disease. To our knowledge, this is the first discovery of genetic variation in RNF139 gene, which provides an approach for the study of HBs disease at the gene level. Meanwhile, we are trying to gather more HBs samples for sequencing detection. Our findings suggested that the genetic test should be performed for early detection of HBs in other family members when HBs patient is found in the family.

## Conclusion

We report a novel mutation of RNF139 p.Q650R (c.A1949G) in a family diagnosed with HBs. Based on the important role of RNF139 gene in the development of cancer, the pathogenicity of the mutation, and the clinical manifestation of the patient (phenotypic characterization), we hypothesized that this mutation could play a pathogenic role in the mechanism of HBs progression.

## Ethics Statement

This study was approved by the ethics committee of Hunan Brain Hospital. A written informed consent was obtained from the patients for the publication of this case report. No investigation or intervention was performed outside routine clinical care for these patients.

## Author Contributions

PY and LiaL conceived the idea, revised all the literature, and wrote the manuscript. BL, LinL, and HxH collected the clinical data. KL analyzed and interpreted brain imaging. HL and WZ performed the NGS and Sanger sequencing. HyH, SZ, and FL contributed to the revision of the manuscript, read, and approved the submitted version.

### Conflict of Interest Statement

The authors declare that the research was conducted in the absence of any commercial or financial relationships that could be construed as a potential conflict of interest.
